# Effect of video-based information on preoperative State trait anxiety inventory scores in adult patients presenting for elective caesarean section: a randomized controlled trial

**DOI:** 10.4314/ahs.v22i3.14

**Published:** 2022-09

**Authors:** Timothy Kanyeki, Vitalis Mung'ayi, Rajpreet Bal, David Odaba

**Affiliations:** Department of Anaesthesia, Aga Khan University, East Africa

**Keywords:** Video-based information, anxiety inventory scores, elective caesarean section

## Abstract

**Background:**

Preoperative anxiety is a common occurrence in patients presenting for surgery with a reported incidence of up to 80%. Increased preoperative anxiety has been associated with increased morbidity. Provision of information relating to surgery and anaesthesia to patients has been proven to have benefit in allaying anxiety. However, the best format of information dissemination remains unknown.

**Objective:**

To determine the effect of video information in addition to the pre-anaesthetic review on the mean preoperative State anxiety inventory (STAI-S) score in adult patients presenting for elective caesarean section under spinal anaesthesia at Aga Khan University Hospital, Nairobi (AKUHN), and to determine the prevalence of preoperative anxiety in the obstetric population presenting for elective caesarean section at AKUHN.

**Methods:**

Thirty-seven adult patients booked for elective caesarean section under spinal anesthesia were randomly assigned to one of two groups. In the study arm; a video was shown to the participants in addition to the standard pre-anaesthetic review. In the control arm the participants only had a standard pre-anaesthetic review.

**Results:**

The mean STAI-T score in the sampled population was 45.64 (SD 5.625). The mean baseline STAI-S score was 46.32 (SD 4.911). There was no statistically significant difference in change in STAI score between the video and control arms (p>0.05).

**Conclusion:**

On the basis of this study among this population, there was no benefit demonstrated from the use of an information video about spinal anaesthesia on anxiety levels in obstetric patients presenting for a first time spinal.

## Introduction

Anxiety has been described in the Diagnostic and Statistical Manual for Mental Disorders (DSM) as ‘subjective feeling of unease, discomfort, apprehension or fearful concern accompanied by autonomic and somatic manifestations. Anxiety is a normal, emotional, reasonable and expected response to real or potential danger’.^1^ The DSM criteria lacks a specific classification for preoperative anxiety which can be described as a form of anxiety that presents before a surgical or anaesthetic procedure. Anxiety has also been described by Spielberger and colleagues to exist in two forms, State anxiety and Trait anxiety.^2^ State anxiety is a ‘temporal cross section in the emotional stream of life of a person consisting of subjective feelings of tension, apprehension, nervousness and worry and activation of the autonomic nervous system. Trait anxiety is the relatively stable individual differences in proneness to perception of stressful situations as threatening or dangerous and in disposition to respond to such situations with more frequent and intense elevations in state anxiety”.

**Table 1:** Summary of study findings

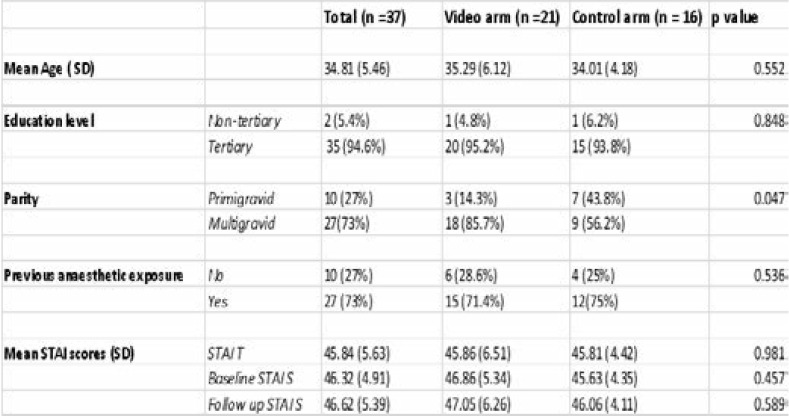

Comparisons were performed using Students t-test.

Pre-operative anxiety remains a common and widely expected occurrence among patients presenting for elective and emergency surgery. The incidence of significant preoperative anxiety has been quoted in literature as being between 60–80% across various populations in the West, Asia and even in the African population.^3^,^4^–^6^

Increased anxiety prior to surgery has been shown to cause detrimental physiological changes leading to poorer anaesthetic and surgical outcomes. It may also lead to refusal of certain interventions and even cancellation of planned surgery. It is clear therefore that anxiety can impact negatively on overall patient satisfaction with perioperative care.^7^–^10^

Anxiety in the obstetric population has been shown to be fairly common with incidences of up to 70%.^10^,^11^ Machaya et al found that 69% of the sampled population in two maternity centres in Zimbabwe had baseline scores that were significant for high anxiety levels. In parturients, anxiety has been attributed in part to factors arising from the pregnancy including worries about the means of delivery and thewellbeing of child following birth.^11^

Maternal anxiety has been postulated to lead to uterine vasoconstriction due to activation of the sympathetic system, therein leading to fetal stress. Teixeira et al showed an association between anxiety during pregnancy and the uterine resistance index.^12^ It has also been shown that patients with high baseline sympathetic activation experience more profound hypotension following neuraxial blockade.^13^ Despite the fact that anxiety about the conduct and outcome of spinal anaesthesia is a recognized problem in obstetric patients, there are currently no interventions to address this in our setting. Moreover, preoperative adjunctive sedative medications are generally avoided in this population. It has been shown that lowering preoperative anxiety leads to better maternal satisfaction following elective caesarean section.^7^

Apart from pharmacologic means of allaying anxiety, other modalities have been studied. Provision of adequate information and patient education in the perioperative period has been shown to be effective. As early as 1963, Egbert LD et al showed the benefit of a review before surgery in allaying anxiety when compared to a sedative (pentobarbital) administered one hour preoperatively. The sedative side effects of pentobarbital were avoided while still accruing benefits of reduced anxiety.^14^ Jafar et al showed marked reduction in incidence of anxiety among patients who had visited the anaesthesia clinic (49%) versus those who had not (86%).^4^

More recently, Kamau et al sought to evaluate the value of a preoperative anaesthesia consult in the clinic compared to the ward with regards to change in anxiety scores. Overall, it was found that the preoperative consult alone did not adequately address patient anxiety to cause a clinically significant change in the scores.^15^ This leaves a gap in the patients' care that is often overlooked.

The value of multimedia means of information including audio recordings, videos, and the internet (e-information) in allaying preoperative anxiety has been assessed in multiple studies. Results from these studies have been conflicting. The ideal method of delivering information therefore still remains unknown. This study intended therefore to assess the effect of the addition to the standard pre-anaesthetic review of a short video depicting the conduct of a spinal anaesthetic on the preoperative anxiety in obstetric patients. The aim of our study was to determine the effect of video information on preoperative State Anxiety scores in adult patients presenting for elective caesarean section under spinal anaesthesia. Our research question was: what is the effect of video information on preoperative State Anxiety scores in adult patients presenting for elective caesarean section under spinal anaesthesia. We hypothesized that there is no effect of video information on change in mean anxiety scores from baseline in adult patients presenting for elective caesarean section under spinal anaesthesia. Our primary objective was to determine the effect of video information on the mean preoperative State Anxiety scores in adult patients presenting for elective caesarean section under spinal anaesthesia. Our secondary objectives were to to determine the prevalence of preoperative anxiety in the obstetric population presenting for elective caesarean section, and to determine the effect of age, level of education, parity and exposure to previous anaesthetic on change in mean preoperative anxiety scores.

## Methods

**Figure 1:**
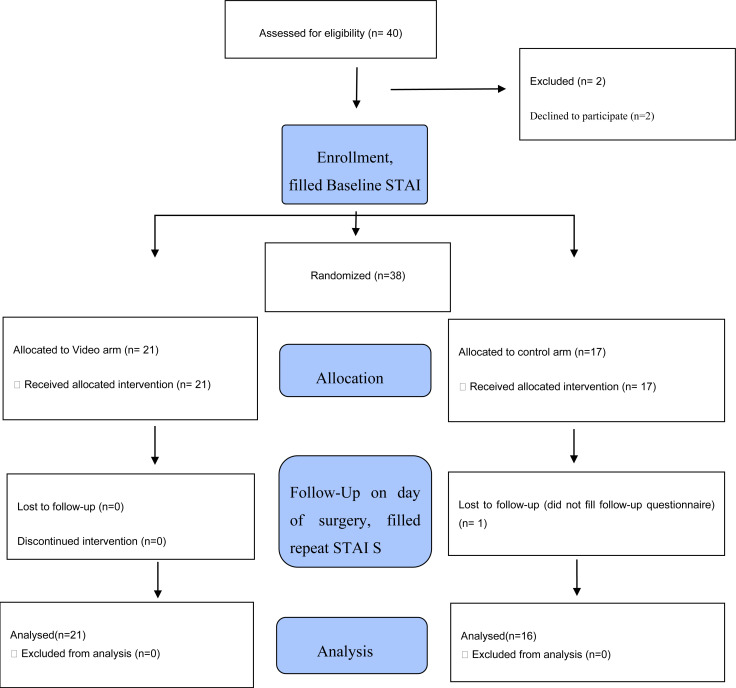
Consort flow diagram of patient distribution

Approval to conduct the study was sought and obtained from the Aga Khan University Research Ethics Committee prior to initiating the study. The study was carried our between November 1 2018 and June 30 2019. Participant flow diagram is shown in figure 1. This was a prospective, superiority, unblinded, randomized controlled trial. The study was conducted at the Aga Khan University Hospital, Nairobi. This is a private university hospital that offers postgraduate medical education programs in various disciplines. It is situated in Nairobi City and therefore serves a cosmopolitan patient population representative of varied ethnic backgrounds. All patients over 18 years who were booked for elective caesarean section and had consented for spinal anaesthetic were included in the study. Reasons for exclusion from the study were:

1. Refusal to participate in the study
2. Previous neuraxial anaesthetic
3. Patients unable fill the questionnaire including those unable to read and/or write, non-English speaking, or with markedly poor eyesight
4. Patients with factors that predispose to high anxiety levels including high risk pregnancies, concurrent uncontrolled thyroid disorder, and history of psychiatric illness
5. Use of psychotropic medications that may give erroneous anxiety scores
6. Patients premedicated with an anxiolytic prior to filling questionnaire

Sample size calculation was based on results from a study by Dias that had a similar study methodology involving assessment of change in STAI scores after an intervention.^16^ Assuming a 5% level of significance and a power of 80%, a sample size of 18 was found to be sufficient to show the above difference between patients who were shown a video and those who receive standard care of practice at Aga Khan University Hospital, Nairobi. Accounting for a 20% drop out rate gives a total of 22 patients per arm for a total of 44 patients. Study participants were enrolled after screening to determine eligibility and obtaining informed consent for the study. Participants were screened during the routine pre-operative anaesthesia assessment session in the anaesthesia clinic up to a maximum of four weeks prior to the date of surgery. This is in keeping with the validity period of a signed consent as per hospital guidelines. All potential participants were consecutively sampled. They then received verbal and written explanation on the purpose and procedure of the study from the reviewing anaesthetist and written informed consent sought. A baseline scoring of State and Trait anxiety was then done using the self-administered STAI questionnaire. The STAI-S questionnaire was administered before the STAI-T as recommended by the developers of the tool. Randomization into either arms then followed.

The STAI-S questionnaire was again administered on the day of surgery. This was done at least 30 minutes prior to surgical time in the wards.

A random allocation sequence was generated by a statistician using a computer algorithm and sealed opaque envelopes were serialized to correspond to the aforementioned random allocation sequence. These envelopes were placed at the anaesthesia clinic. Once informed consent was given, the clinician picked an envelope and opened it in the presence of the patient. The clinician then explained to the study participant to which arm they had fallen. Only those randomized into the video arm were shown the video on a tablet. They were allowed to review the video as required and follow-up questions addressed. A copy of the video was offered on compact disc for review at the participant's leisure.

The video was a 7.55-minute film with actors drawn from among residents and theatre staff. It was filmed in the university hospital theatres to increase patient familiarity with the surroundings. It was edited to incorporate a running commentary of events and an overview of common risks and the expected outcomes of a successful block. It addressed the common patient concerns regarding spinal anaesthesia identified from review of literature.

Patient biodata was collected by the recruiting anaesthetist using a standard biodata form. Baseline and follow-up STAI S and T scores were compiled from the filled questionnaires out of 80 as per the accompanying manual. These data were entered into SPSS for analysis. Demographic characteristics were expressed as: Age (years), parity (primigravida, or multigravida), previous exposure to anaesthetic, (Yes or No), and level of education (tertiary or non-tertiary).

Data analysis was done using the SPSS version 20 with the assistance of a statistician, and the results presented as tables and charts. P < 0.05 was considered statistically significant for all data. Continuous data were described in terms of means with standard deviation, and percentages as appropriate. Comparison of means was done using Student's t and paired sample t tests. Fisher's exact test was used to investigate the association of the independent factors and the anxiety scores.

## Results

Forty patients were found eligible between November 2018 and June 2019. Of these, two were excluded during enrolment after declining to participate. 21 were randomized into the video arm and 17 into the control arm. One patient was lost to follow up and subsequently excluded from analysis as shown in figure 1.

The median age was 35 and there was no difference in age distribution between the arms. 94.6% of study participants had a tertiary level of education, 64.9% of which was at graduate level. 27% of participants were primigravid and 73% multigravid. 73% of the participants had had a previous anaesthetic exposure and none was a regional technique. The mean STAI-T score in the sampled population was 45.84 (SD 5.63). The mean baseline STAI-S score 46.32 (SD 4.91) while the mean repeat STAI-S score was 46.62(SD 5.39). There was no statistically significant difference in the STAI scores between the video and control arms. These findings are summarized in table 1 below. In this sampled population, it was found that 29.7 % of patients had moderate Trait anxiety, 62.2 % had high anxiety. 21.6% had moderate baseline State anxiety, 70.3% had high anxiety. 32.4% had moderate follow up State anxiety, 59.5% had high anxiety.

The Shapiro Wilk test was used to determine normality in the distribution of the continuous variables. The results are summarized in table 2 below. All data were normally distributed.

**Table 2:** Shapiro Wilk test for normality

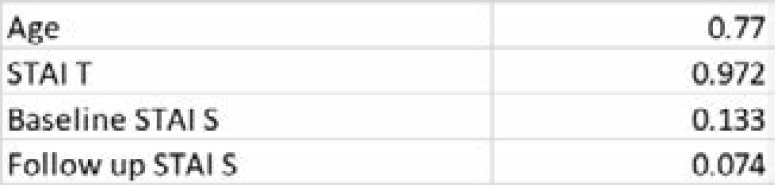

The primary objective in this study was to determine the difference in reduction of anxiety quantified by the change in the mean STAI S score between the baseline and the follow up STAI S scores. The mean baseline STAI S score in the video arm was 46.86 (range 36 – 61) and in the control arm was 45.63 (range 36 – 52). The mean follow up STAI S score in the video arm was 47.05 (range 39 – 60) and in the control arm was 46.06 (range 40 – 54). There was no statistical significance found in the comparison of the difference in the change of mean STAI S score between the arms as summarized in table 3. Finally, the influence of the independent factors age, level of education, parity, and previous anaesthetic exposure on the change in the STAI S score was investigated. It was found that there was no effect from these independent variables on the change in the mean score between the arms except for the STAI-T with a p value of 0.001. This is summarized in table 4.

**Table 3:** Comparison of the change in mean STAI S scores

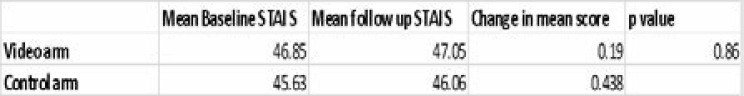

Comparison of means were performed using Students t-test.

**Table 4:** Influence of independent factors on change in STAI S scores

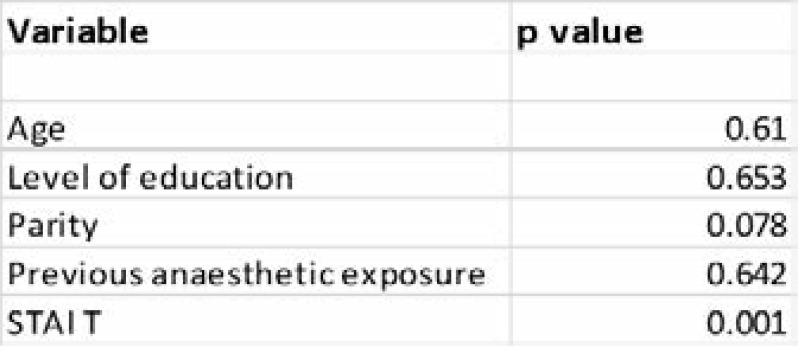

Categorical comparisons were performed using a 2-sided Fisher's exact test.

Continuous comparisons were performed using Students t-test

## Discussion

There was no difference in patient demographics between the study arms. The mean age in the cohort was 34.8 years. This reflects an older cohort of obstetric patients presenting to our hospital compared to public facilities. A survey in Nyanza province in Kenya of births between 1988 – 1993 showed that only 15.6% of mothers were aged 35 years and above.^17^

In both groups, more than 90% of study participants had a tertiary level of education which corresponds to at least 12 years of school in the Kenyan education system. A majority had a graduate degree. This as well reflects a more educated population that is served by our hospital compared to public facilities. A survey in Nyanza province showed that only 17.5% of mothers had level of education beyond primary school. 17

In this study, the main findings were that there was no statistically significant difference in change in STAI-S scores taken during clinic review and preoperatively when an information video was shown after routine anaesthetic assessment. The null hypothesis was therefore accepted. This is similar to findings from previous published works by Salzwedel et al who found no benefit in change of anxiety levels from the use of an anaesthesia information video in non-obstetric patients.^18^ Eley et al as well found that a video depicting a neuraxial anaesthetic shown during routine anaesthesia review did not lead to reduction in patient anxiety in elective caesarean section.^19^

It was also found that there were no statistically significant differences in the baseline and repeat STAI-S and the STAI-T scores between the two arms. Furthermore, there was no statistically significant effect of either age, parity, level of education, or previous anaesthetic exposure on baseline scores or change in scores. However, post hoc analysis showed the STAI-T score to have an effect on the STAI-S score, meaning that a participant's propensity to anxiety correlated well with their state anxiety score. Further studies should consider whether more intervention is required while managing patients coming for anaesthesia and surgery with a high baseline STAI-T score.

There was a 10% reduction in the prevalence of high anxiety between baseline and repeat STAI S scores (70.3% to 59.5%) but no significant change in the mean anxiety scores in both arms. It was also noted that there were wide ranges in the anxiety scores within the groups, more so in the video arm. This could be explained by a skewing of the scores by outliers coupled with the small sample size which would have reduced the effect of the intervention.

The response to details regarding aspects of surgery and anaesthesia could possibly vary from patient to patient. It is likely that some patients fair better with fewer details, and would paradoxically have had higher anxiety scores following an intervention such as this information video. Ng SK in a study of basic versus detailed information for dentoalveolar surgery under local anaesthetic showed that details of the procedure benefited the low-trait anxiety participants rather than the high-trait anxiety. This might perhaps explain the skew in some of the data in this study.^20^

This study found a prevalence of 70% for high anxiety in the sampled population. Studies in similar populations have shown prevalence of 75% in 2 maternity centres in Zimbabwe in recent study by Machaya et al, and 80% by Dias et al. 11,16

The mean STAI-S and STAI-T scores in the sampled population were 46.32 (SD 4.911) and 45.84 (SD 5.625) respectively. This is comparable to results by Kamau et al who reported on anxiety levels during anaesthesia reviews of patients in the clinic and in the wards at AKUHN. They sampled patients from all surgical disciplines and found a mean score of 46.86 in the patients reviewed in clinic.^18^ Mean anxiety levels in the obstetric population sampled in this study are therefore no different from the general population at AKUHN.

A reduction of 8 to 10 points in the STAI scores had been hypothesized to show significance from the intervention in this study. Although the mean change in score was not significant, analysis of the individual samples shows wide ranges that could possibly have shown effect in a larger sample size.

It is likely that the urban and well educated population from which our cohort was sampled received information about anaesthetic techniques from other sources including social media, which could have affected the anxiety scores.

## Strengths of the study

This was a randomized study with an appropriate control group to compare the effect of the intervention. It was the first study of its kind in the obstetric population in our setting. There was use of a tool validated for measuring of anxiety in the obstetric population. The trait anxiety scores correlated with the state anxiety scores showing that the tool was applied appropriately. The comparison of the change of the STAI score from baseline as opposed to a single score was an added strength.

## Weaknesses of the study

This study had some limitations. The exclusion criteria may have led to selection bias as majority of patients presenting to AKUHN for elective caesarean section had had a previous regional anaesthetic and were therefore not included. It is likely that although the study was adequately powered, a larger sample size would have given a larger change in mean scores as evidence by the significant range. The population served by the AKUHN, a private facility, is unlikely to be comparable to other facilities and as such the results are not generalizable to the rest of the population in the country.

Although the study protocol endeavored to avoid cross-contamination of the groups. The participants enrolled into the intervention arm were asked to avoid sharing the video with other persons. However, as there was no way to control for this, this could have been a confounding factor where it was not disclosed to the reviewing anaesthetist. It is also possible that the study participants could have opted to look up other available material following enrollment and this could have affected scores in either group.

## Conclusion

On the basis of the results of this study, there is no demonstration of benefit from an information video about spinal anaesthesia in reducing anxiety scores in obstetric patients presenting elective caesarean section. The prevalence of high anxiety in the sampled population was 70%. Further, there was no demonstrated effect of age, level of education, parity, or history of previous anaesthetic on change in anxiety scores in this population.
